# Structural Based Analyses of the JC Virus T-Antigen F258L Mutant Provides Evidence for DNA Dependent Conformational Changes in the C-Termini of Polyomavirus Origin Binding Domains

**DOI:** 10.1371/journal.ppat.1005362

**Published:** 2016-01-06

**Authors:** Gretchen Meinke, Paul J. Phelan, Jong Shin, David Gagnon, Jacques Archambault, Andrew Bohm, Peter A. Bullock

**Affiliations:** 1 Department of Developmental, Molecular and Chemical Biology, Tufts University School of Medicine, Boston, Massachusetts, United States of America; 2 Sackler Institute of Graduate Biomedical Sciences, New York University School of Medicine, New York, New York, United States of America; 3 Institut de Recherches Cliniques de Montreal (IRCM), Montreal, Quebec, Canada; 4 Department of Biochemistry and Molecular Medicine, Universite de Montreal, Montreal, Quebec, Canada; University of Wisconsin-Madison, UNITED STATES

## Abstract

The replication of human polyomavirus JCV, which causes Progressive Multifocal Leukoencephalopathy, is initiated by the virally encoded T-antigen (T-ag). The structure of the JC virus T-ag origin-binding domain (OBD) was recently solved by X-ray crystallography. This structure revealed that the OBD contains a C-terminal pocket, and that residues from the multifunctional A1 and B2 motifs situated on a neighboring OBD molecule dock into the pocket. Related studies established that a mutation in a pocket residue (F258L) rendered JCV T-ag unable to support JCV DNA replication. To establish why this mutation inactivated JCV T-ag, we have solved the structure of the F258L JCV T-ag OBD mutant. Based on this structure, it is concluded that the structural consequences of the F258L mutation are limited to the pocket region. Further analyses, utilizing the available polyomavirus OBD structures, indicate that the F258 region is highly dynamic and that the relative positions of F258 are governed by DNA binding. The possible functional consequences of the DNA dependent rearrangements, including promotion of OBD cycling at the replication fork, are discussed.

## Author Summary

A conserved feature of Polyomavirus T-antigens is a phenylalanine situated at the C-termini of their origin-binding domains (OBDs). Using the T-antigen encoded by JC virus, we have investigated why this residue is critical for viral DNA replication. The studies presented herein establish that the consequences of this mutation are limited to the interface formed by the docking of the phenylalanine containing C-terminal pocket region of the OBD with the multifunctional A1/B2 region. Related studies indicate that the conformation of the C-terminal region of the OBD is altered by DNA binding. These observations suggest a model whereby cycling of the OBDs within the hexameric spiral structure at the replication fork is promoted by DNA binding.

## Introduction

There are presently fourteen known human polyomavirus family members [1, 2]. Reasons for interest in these viruses include the diseases they are associated with, particularly in immunocompromised individuals [3–5]. As examples, JC virus (JCV) causes the often fatal demyelinating disease Progressive Multifocal Leukoencephalopathy (PML) ([6]; reviewed in [7]); Merkel cell polyomavirus causes Merkel cell carcinoma, a rare but highly aggressive skin cancer [8, 9] and BK polyomavirus causes BK nephropathy [10, 11]. Further interest in polyomaviruses stems from the profound insights they have provided into basic cellular process, such as DNA replication (e.g., [12–17]) and the mechanisms that underlie cellular transformation (e.g., [3, 18–20]).

Polyomaviruses have small double stranded DNA genomes [14] that contain a regulatory region that is termed the non-coding control region (NCCR). The NCCR contains the origin of replication as well as the promoter and enhancer elements (reviewed in [21, 22]). An additional feature of polyomavirus genomes is the "early region" that encodes several proteins, including the large T-antigen (T-ag; reviewed in [15, 23, 24]). T-ag is the only virally encoded protein needed for replication; therefore, it has been the target of numerous studies designed to understand its multiple roles during the duplication of the viral genome (reviewed in [12, 15, 25]). For example, polyomavirus T-ag's have been the focus of a number of recent structural studies (reviewed in [25, 26]). These structural studies have provided critical insights into the initiation process, such as establishing how the GAGGC pentanucleotides in the polyomavirus replication origins are recognized by the origin binding domains (OBD) within T-ag [27–32]. Related studies, conducted with the SV40 T-ag OBD, suggest that following site-specific DNA binding, the OBDs undergo rearrangements, including "spiral formation" [32–35]. Additional experiments have revealed the multiple roles played by the T-ag helicase domains during origin melting [36–38], oligomerization [39, 40] and helicase activities ([39, 40]). Based on these studies, models depicting T-ag's multiple roles during the initiation of SV40 DNA replication have been proposed (e.g., [25, 26, 41]).

To further our understanding of the initiation of polyomavirus DNA replication, we recently initiated structural studies of JCV T-ag. In particular, we solved the structure of the JCV T-ag OBD [42]. One of the interesting findings of the JCV T-ag OBD structure was the presence of a C-terminal "pocket". This structure also revealed that the pocket serves as the binding site for T-ag residues from the A1/B2 loops situated on a neighboring OBD subunit [42]. The binding of the A1/B2 loops to the pocket was of interest given that the initial function of the A1/B2 loops is to site-specifically bind the pentanucleotides in the core origin ([43, 44]; reviewed in [45]). These observations provided further evidence that the A1/B2 loops are multifunctional (reviewed in [26]) and that the interaction of the A1 & B2 loops with the pocket is a critical step that takes place at later stages of the initiation process (e.g., during the oligomerization of T-ag on the core origin ([42]; reviewed in [26]).

Additional evidence for the hypothesis that the pocket in the OBD plays a critical role during JCV replication was derived from studies of the JCV T-ag F258L mutant [42]. The F258L "pocket mutation" had no effect on levels of T-ag expression, but for unknown reasons it inactivated T-ag dependent JCV replication [42, 46]. Therefore, given our interest in the JCV T-ag OBD pocket, and its role(s) during JCV DNA replication, we elected to examine the structural basis for the inactivation of JCV replication by the F258L T-ag mutation. The results of these experiments, presented herein, prompted us to examine the dynamic properties of the C-terminal regions of polyomavirus T-ag OBDs. These structure-based analyses indicate that the C-terminal region of the SV40 T-ag OBDs move as a function of DNA binding. The possible biological consequences of these DNA dependent movements are discussed.

## Materials and Methods

### 1. Construction of vectors

An expression vector encoding the JCV T-ag OBD (residues 132–261), that was termed pGEX1λT JC-OBD, was previously described [42]. Using the Quikchange Kit (Agilent Technologies), JCV-OBD residue Phe 258 was mutated to Leu. The oligonucleotides used for the mutagenesis were 5’-GGCCTTAAGGAGCATGACCTTAACCCAGAATAATCG-3’ (the mutated base is underlined) and its complement. The resulting plasmid was termed pGEX1λT JCV-OBD-F258L. The same mutation had been previously introduced into full-length JCV T-ag [42], using the Quikchange Kit, plasmid pCMVneo JCVT-ag and the oligonucleotides listed above. The resulting plasmid was termed pCMV-JCVT-ag F258L. DNA Sequencing at the Tufts University Core Facility (TUCF) confirmed the sequence of the F258L mutants in plasmids pGEX1λT JCV-OBD-F258L and pCMV-JCVT-ag F258L.

### 2. Sequence alignments

Sequence alignments were performed with the program Clustal Omega at the EMBL-EBI website [47]. The aligned sequences were displayed using the program JalView [48].

### 3. Protein purification

The wild type JCV T-ag OBD was purified using a previously described protocol [42]. The JCV T-ag OBD F258L mutant protein was purified from BL21 cells using the identical procedures used to purify the wt JCV T-ag OBD [42]. Once purified, the JCV wt and F258L OBD proteins were stored at -80°C in storage buffer (20 mM Tris pH 8.0, 50 mM NaCl, 10% glycerol, 1 mM EDTA, 0.1 mM PMSF and 5 mM DTT).

### 4. Immunofluorescence

The subcellular localization of the F258L JCV T-ag mutant within C33A cells was determined by immunofluorescence ([49] and references therein. A detailed protocol describing the steps needed to detect JCV T-ag within C33A cells was previously published [46]). T-ag was detected using the Pab 416 monoclonal Ab (Santa Cruz Biotechnology) and a secondary goat anti-mouse antibody, conjugated with Alexa 488 (Life technologies). The cells were visualized using a Zeiss Axiovert 200M microscope and the data were analyzed using the OpenLab software package from Perkin Elmer.

### 5. X-ray crystallography

#### a. Crystallization

Crystals of the JCV T-ag OBD F258L mutant were grown in hanging drops at 18 C by vapor diffusion over a 1 mL reservoir in a Linbro plate (Hampton Research Inc) by mixing 1 ul of the protein (at 10 mg/ml in storage buffer) with 1 ul of the reservoir solution (0.1 M Tris pH 8.5, 25–35% Peg 6000). The crystals were harvested by transferring them to a cryogenic solution (0.085 M Tris pH 8.5, 29.75% Peg 6000, 15% ethylene glycol) using a cryo-loop, then flash-freezing and storing the crystals in liquid nitrogen prior to X-ray data collection.

#### b. X-ray data collection and structure solution

The final X-ray data were collected at 100 K at NSLS Beamline X29 (Brookhaven National Laboratory, NY). The X-ray data were processed with HKL2000 [50] to a resolution of 2.7 Å. The details of the data collection and refinement are summarized in Table 1.

**Table 1:** X-ray Data collection and refinement statistics.

Crystal Name	JCV OBD F258L
PDB ID	5CYN
**Data collection**	
Wavelength (Å)	1.075
Space group	I _41_
**Cell dimensions**	
*A*, *B*, *C* (Å)	103.54, 103.54, 34.34
α, β, γ (°)	90.00, 90.00, 90.00
Resolution (Å)	50.00–2.7 (2.8–2.7) *
*R*_sym_ or *R*_merge_	0.12 (0.66)
*I* / σ*I*	37.4 (2.7)
Completeness (%)	99.6 (96.6)
Redundancy	15.4 (7.1)
**Refinement**	
Resolution (Å)	50–2.7
No. reflections	5163
*R*_work_ / *R*_free_	19.5, 22.7
No. atoms	1043
Protein	1032
Ligand/ion	4
Water	7
*B*-factors	71.0
Protein	71.3
Ligand/ion	61.8
Water	53.0
**R.m.s. deviations**	
Bond lengths (Å)	0.009
Bond angles (°)	1.190

*Values in parentheses are for highest-resolution shell.

The JCV F258L OBD mutant crystallized in the space group I41, with one molecule in the asymmetric unit. This crystal form is virtually identical to the wt JCV OBD crystal form (PDB ID = 4LIF). Therefore, the 4LIF structure was used as a search model for the F258L mutant crystal form. The F258L mutant structure was solved by molecular replacement using the program PHASER ([51]; available within the CCP4 suite [52]). Both the program Refmac5 [53], within the CCP4 suite, and the program Phenix [54] were used to refine the structure at different stages. The molecular graphics program Coot [55] was used for manual rebuilding between successive rounds of refinement. The program PDBREDO [56] was used to improve the geometry prior to deposition of the coordinates. The refined coordinates have been deposited to the Protein Data Bank (PBD) and given the accession code 5CYN.

#### c. Structural analyses and molecular visualization

The structure of the JCV T-ag OBD F258L mutant was analyzed using the program PDBSUM [57]. The crystallographic interface formed between the pocket residues and the A1/B2 loops was analyzed using the program PISA [58]. The molecular graphics program PyMOL [59] was used to generate molecular figures. Superpositions were performed using the program SSM [60].

### 6. Isothermal Titration Calorimetry (ITC)

The ITC studies were conducted with a VP-ITC calorimeter (MicroCal, Northampton, MA). Prior to conducting the ITC studies, the dsDNA oligonucleotide and the JCV T-ag OBD proteins (both wt and the F258L mutant) were buffer-exchanged, using PD-10 columns (GE Healthcare), into 10 mM Sodium Phosphate, pH 7.0, 50 mM NaCl and 5 mM DTT. Protein and DNA concentrations were determined spectrophotometrically, using extinction coefficients calculated with the ProtParam web server and the Integrated DNA Technologies (IDT) website, respectively. The data were analyzed using the Origin software provided by the manufacturer.

### 7. Fluorescence anisotropy DNA-binding assay

Binding isotherms and K_D_ measurements were performed essentially as described [61, 62]. The reactions were performed in 96-well plates (OptiPlate-96 F HB black microplate, Perkin Elmer), in a final volume of 150 μl containing 15 nM of fluorescein-labeled probe and the indicated concentrations of T-ag OBD in buffer containing 20 mM Hepes pH 7.4, 50 mM NaCl, 0.01% NP40 and 0.1 mM DTT. Fluorescence readings were taken on a Victor^3^V 1420 Multilabel HTS Counter (Perkin Elmer) using the 485 nm/535 nm filter sets. Background fluorescence from buffer was subtracted and polarization (P) and anisotropy (A) values were defined as P = (III−I⊥)/(III + I⊥) and A = (III−I⊥)/(III + 2I⊥), where III and I⊥ are the fluorescence intensities recorded in the parallel and perpendicular orientations respective to the orientation of the excitation polarizer. Fluorescein-labeled oligonucleotides were purchased with the fluorophore attached at the 5`end by a six-carbon linker (IDT). Duplex DNA probes were prepared by annealing each fluorescein-labeled oligonucleotide to a complementary oligonucleotide. Apparent K_D_ values were obtained from direct binding isotherms by nonlinear least-squares regression of the data as previously described [62].

## Results

**Fig 1:**
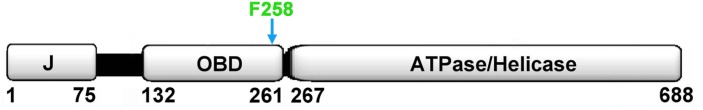
Location of T-antigen residue F258. A schematic of JCV T-ag, showing the relative locations of the major domains and the position of residue F258 at the C-terminus of the JCV T-ag OBD.

**Fig 2:**
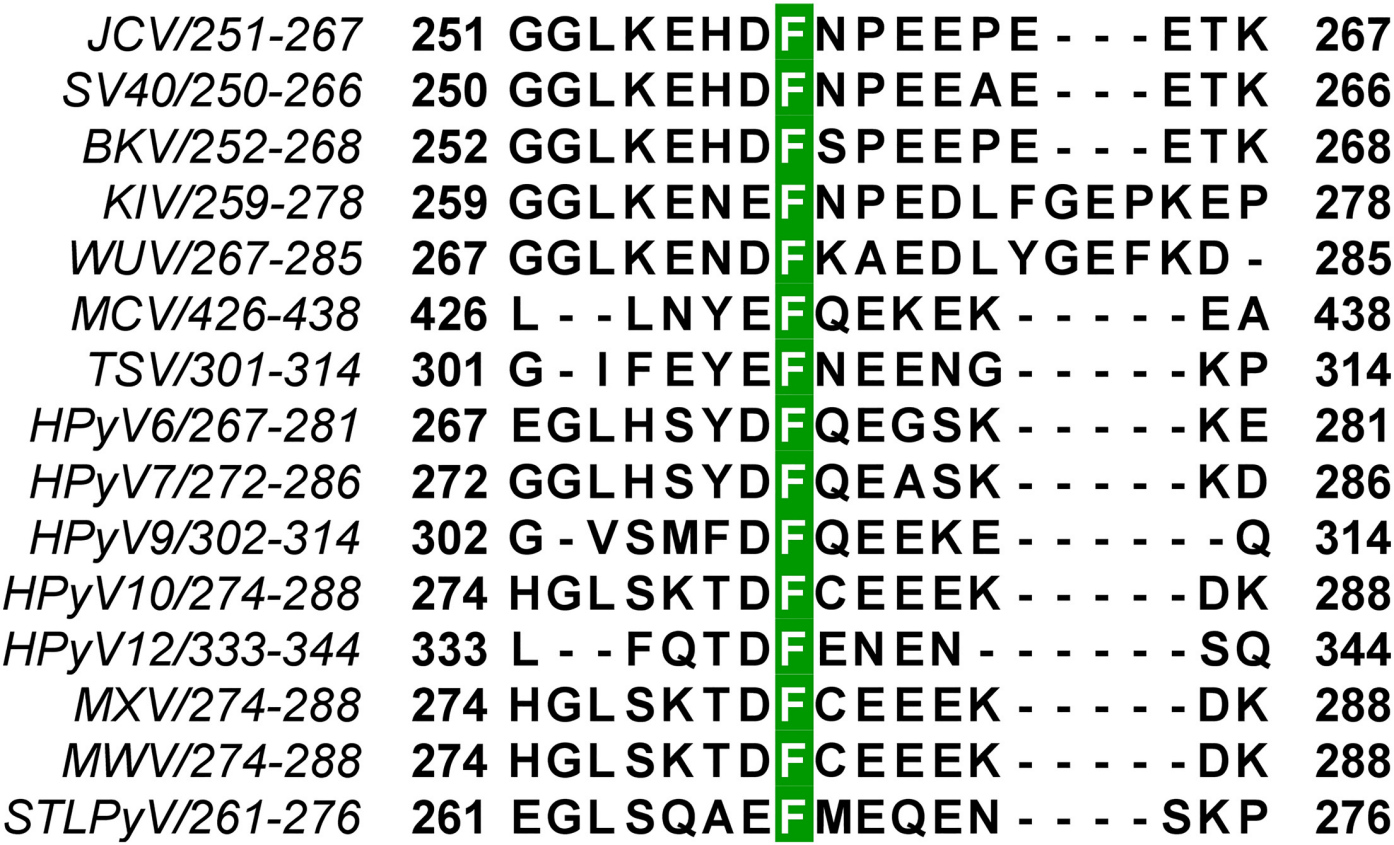
Conservation of polyomavirus T-antigen residue F258. Sequence alignment of the C-terminal regions of the fourteen human polyomavirus T-ag OBDs, along with the SV40 OBD, demonstrating that JCV residue F258 is highly conserved. The accession codes for the amino acid sequences used in the alignment are listed below, with the corresponding polyomavirus name in parentheses: P03070 (SV40), P03071 (BKV), A3R4N4 (KIV), P03072 (JCV), A5HBG1 (WUV), B6DVW7 (MCV), A0A068EVP3 (TSV), D6QWG6 (HPyV6), D6QWI6 (HPyV7), YP_004243706.1 (HPyV9), AFN43007.1 (HPyV10), AGL07668.1 (MWV), AFS65330.1 (MXV), AGH58117.1 (HPyV12) and AGC03170.1 (STLPyV).

JCV T-ag containing the F258L mutation, which is located in the C-terminus of the OBD (Fig 1), was unable to support T-ag dependent JCV DNA replication [42]. Furthermore, all of the human polyomavirus T-ags, as well as SV40 T-ag, have a phenylalanine at the analogous position (Fig 2). Therefore, to address the function(s) of this highly conserved phenylalanine in polyomavirus DNA replication, we initiated studies of the JCV T-ag F258L mutant.

### 1. Subcellular localization of full-length JCV T-ag containing the F258L mutation

We previously characterized, via immunofluorescence, the sub-cellular localization of JCV T-ag in C33A cells; a cell line that supports robust levels of JCV DNA replication [46]. It was concluded that JCV T-ag is predominantly in the nuclei of these cells where it is distributed in a punctate manner. Given that T-ag localization to the nucleus is essential for the replication of polyomaviruses (e.g., [63, 64]), we elected to determine if T-ag containing the F258L mutation is also preferentially localized to the nuclei in C33A cells.

**Fig 3:**
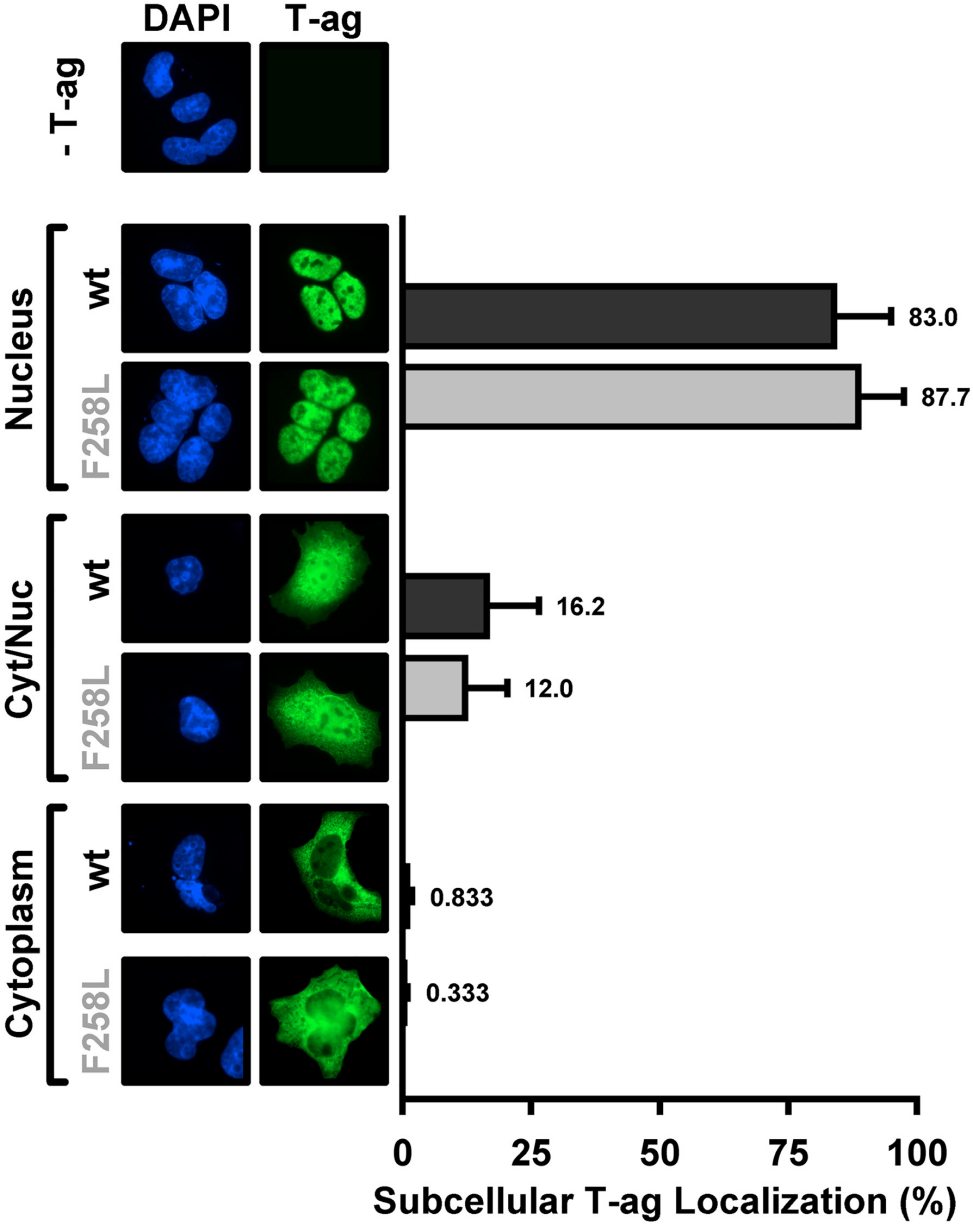
Subcellular localization of the JCV F258L T-ag mutant in C33A cells. Immunofluorescent based comparison of the sub-cellular distribution of wt JCV T-ag, and the F258L mutant (both in green), within replication permissive C33A cells [46]. The cell nuclei were stained with DAPI. The histograms, based on the quantitation of ~ 100 such images, indicate the percentage of T-ag in nuclei, both nuclei and cytoplasm or just cytoplasm for the wt and mutant T-ags.

Inspection of Fig 3 establishes that as with wt T-ag, full-length JCV T-ag containing the F258L mutation is largely in the nucleus (83% vs ~88%; respectively). Similar to wt T-ag, a much lower percentage of cells contain the mutant in both the nuclei and the cytoplasm (~16.2 vs 12%; respectively) or exclusively in the cytoplasm (~ 0.833 vs 0.3%; respectively). Thus, the failure of the JCV T-ag F258L mutant to support viral replication is not due to defects in its subcellular localization.

### 2. The structure of the JCV T-ag F258L OBD mutant

The next step taken to establish the defect caused by the F258L mutation was the determination of the structure of the JCV T-ag OBD F258L mutant. The F258L mutant was purified using previously described methods (materials and methods section). As shown in Table 1, the F258L JCV T-ag OBD crystallized in the I41 space group, diffracted to 2.7 Å and contained one molecule in the asymmetric unit cell.

**Fig 4:**
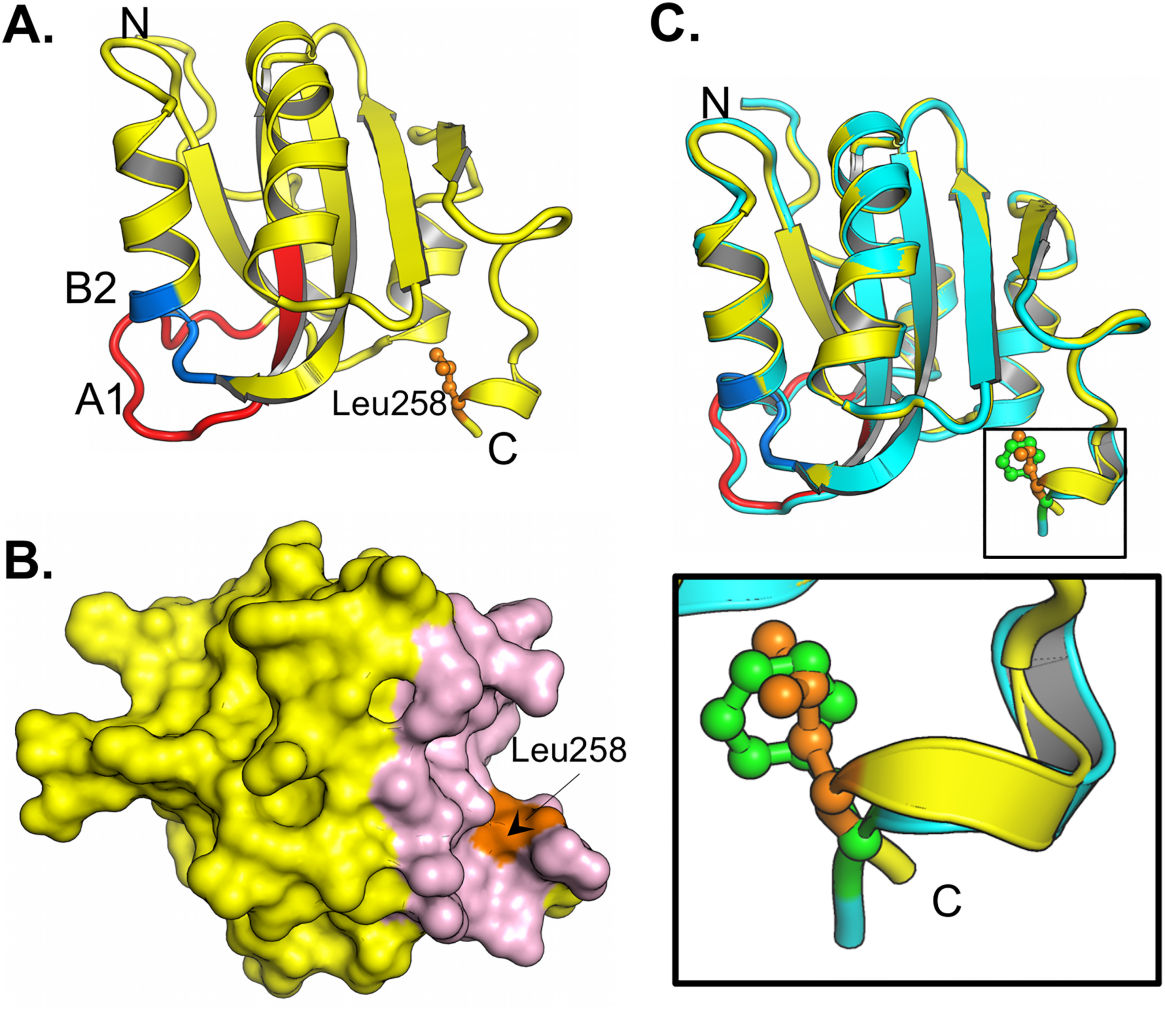
The structure of the F258L JCV T-ag OBD. **A.** A ribbon diagram of the JCV T-ag OBD F258L monomer. The multifunctional A1 (residues 147–155) and B2 (residues 203–207) regions are labeled red and blue, respectively. Residue L258 is shown in orange. The N and C termini are indicated. **B.** A surface representation of the JCV T-ag OBD F258L monomer showing that presence of the C-terminal pocket (in pink) and the location of L258 (in orange). **C.** Superimposition of the F258L mutant (yellow) onto the wt JCV OBD (in cyan). The insert presents a close up of the superimposition of wt F258 (in green) onto L258 (in orange).

The structure of the JCV T-ag F258L OBD mutant is presented in Fig 4A, only residues 133–259 are visible in the crystal structure. As with the wt JCV OBD [42], the topology of the OBD F258L mutant is a five-stranded antiparallel B-sheet sandwiched, on either side, by two helices. The leucine at position 258 is in orange and shown in a ball and stick representation. The multifunctional A1 and B2 loops are shown in red and blue, respectively. The A1 loop is the "DNA free" or "apo-conformation" ([42]; reviewed in [26]). Inspection of Fig 4B establishes that the previously described C-terminal pocket [42] is also a feature of the JCV T-ag OBD F258L mutant. A superposition of the F258L JCV T-ag OBD mutant with the wt JCV OBD ([42]; 4LIF) is shown in Fig 4C; it is apparent that these two structures are nearly identical (RMSD of 0.43 Å over 127 C-alphas). The spatial overlap between residues F258 with L258 is indicated (Fig 4C; (green and orange residues, respectively)). Finally, the structure also revealed that, owing to the relatively small size of leucine, the F258L substitution created a small cavity that was filled by Leu 258 moving closer to the core of the OBD (Fig 4C insert).

### 3. Comparing the ability of the F258L and wt OBDs to site-specifically bind to DNA

The initial function of the A1 and B2 loops is site-specific DNA binding to the viral origin [44, 65, 66]. Spatially, the A1 and B2 loops are relatively close (~ 15 Å) to residue 258 (Fig 4A). Thus, while the two structures are nearly identical (Fig 4C), subtle but significant structural differences may alter the DNA binding specificity of the F258L mutant and thus account for the replication defect associated with this JCV T-antigen mutant. Therefore, we elected to determine if the F258L T-ag OBD is altered in terms of its ability to bind DNA in a site-specific manner.

#### a. Comparing the ability of wt JCV T-ag OBD and F258L mutant to bind the central region of the core origin via ITC

**Fig 5:**
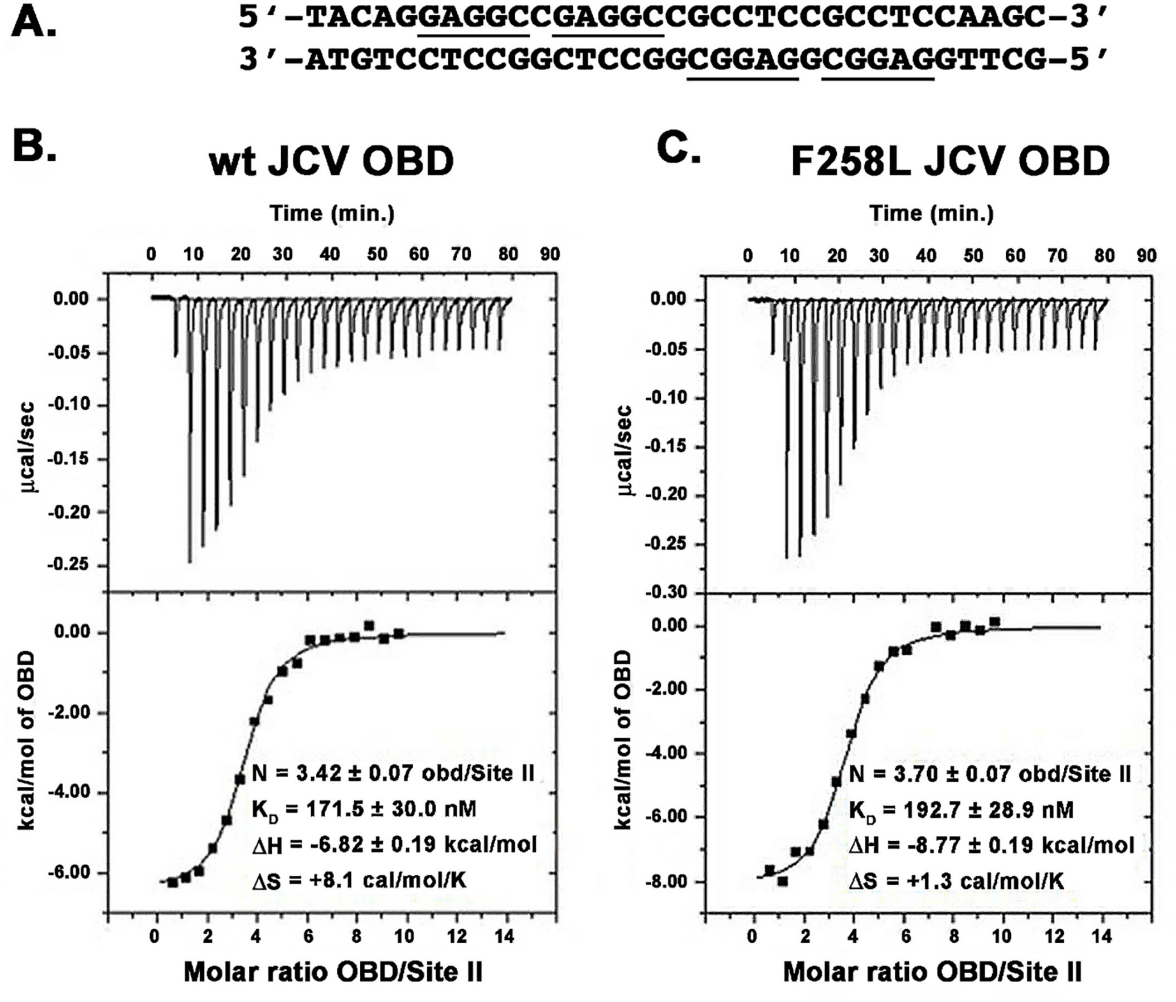
Measuring the affinity of the F258L and wt JCV OBDs to bind to the central region of the core origin via ITC. **A**. The four pentanucleotide containing Site II based duplex oligonucleotide, derived from the JCV origin of DNA replication, used in these studies. The GAGGC sequences are underlined. **B**. An ITC titration for the wt JCV T-ag OBD with DNA. **C**. An ITC titration for the JCV T-ag OBD F258L mutant with DNA. For each study, the calorimetric trace is shown in the top panel; the X-axis is time in minutes, while the Y-axis is power in ucal/s. The binding isotherms are shown in the bottom panel; the X-axis is the molar ratio of the indicated OBD to DNA. The Y-axis is kcal/mol of OBD. Values for K_D_ and stoichiometry (N) are indicated.

Isothermal titration calorimetry (ITC) was used to determine if the F258L mutation compromises the ability of the A1 & B2 loops to site-specifically bind to duplex DNA. The Site II containing duplex DNA used in these studies is presented in Fig 5A. The data presented in Fig 5B and 5C demonstrate that the wt JCV T-ag OBD, and the JCV T-ag OBD harboring the F258L mutation, bind to the duplex oligonucleotide containing four GAGGC pentanucleotides with dissociation constants (K_D_) of 172 nM and 193 nM, respectively. Thus, based on these experiments, there is little evidence that the F258L JCV T-ag OBD mutation alters the affinity of the JCV OBD for Site II. Consistent with a DNA substrate having four binding sites, the N values for the wt and F258L mutant were determined to be 3.42 and 3.7, respectively.

#### b. Comparing the affinity of the wild type and F258L T-ag OBDs for a single pentanucleotide by Fluorescence Anisotropy

**Fig 6:**
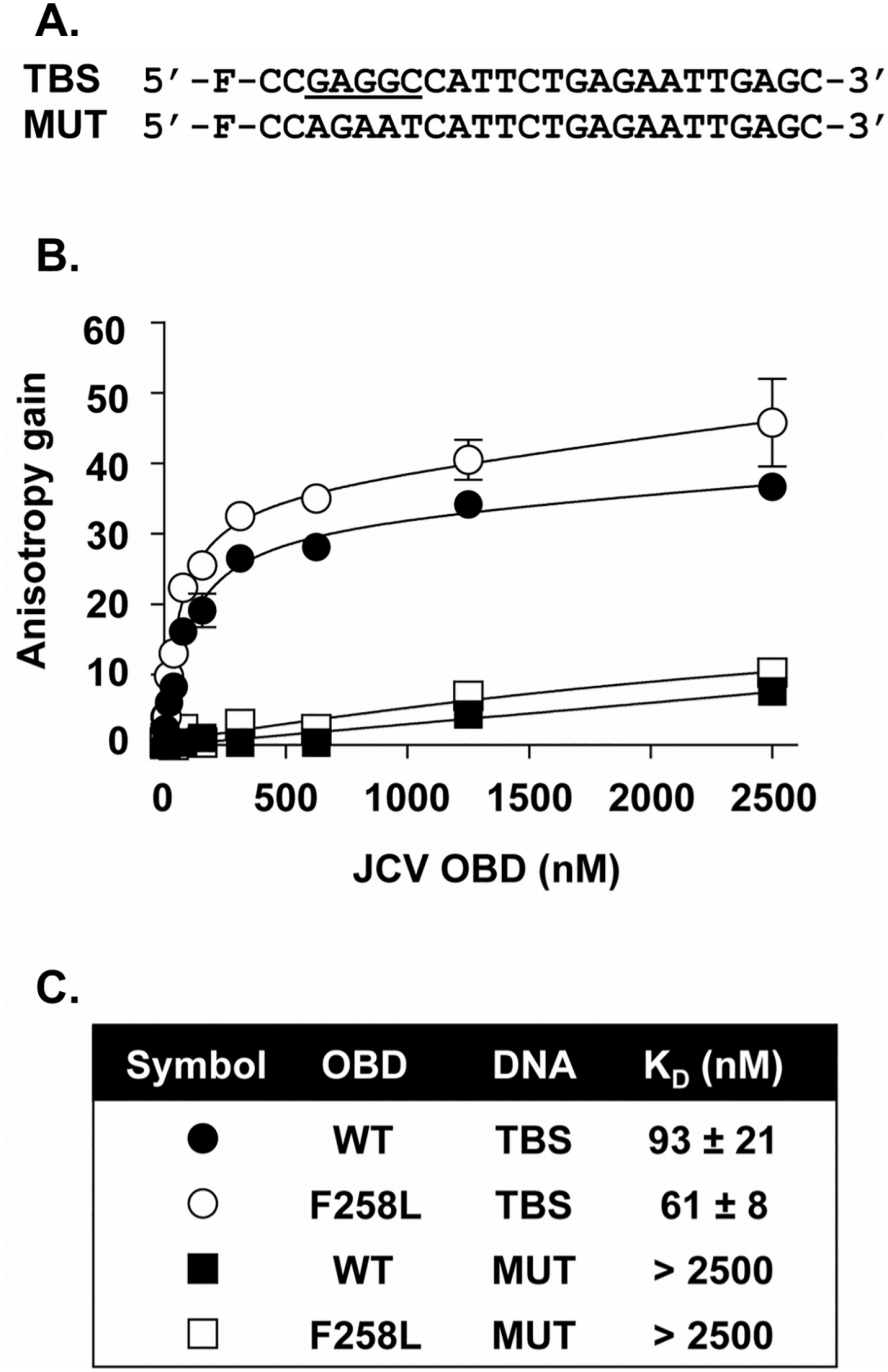
Measuring the ability of the wild type and F258L OBDs to Bind to DNA via Fluorescence anisotropy. **A**. DNA probes used in these experiments. The nucleotide sequences of the probe containing a single T-ag Binding Site (TBS; (GAGGC underlined)), and of the control probe containing a mutated TBS (MUT) are indicated. “F” refers to the position of the fluorescein moiety. **B.** Binding isotherms were acquired with 15 nM of the TBS probe (circles) or control probe (squares), and increasing concentrations of the wild type (wt) OBD (filled symbols) or F258L mutant derivative (open symbols). Each data point is the average of two independent experiments, each performed in triplicate (n = 6). Some of the standard deviations are not visible on the graph as they are smaller than the symbols. **C.** K_D_ values derived from the binding isotherms presented in **B**. Note, the JCV T-ag OBD, and the F258L mutant, bound to the single pentanucleotide containing probe more tightly than to the four pentanucleotide containing probe ((93 ± 21 nM and 61 ± 8 nM (Fig 6C) versus 172 nM and 193 nM (Fig 5B and 5C)). The reason for this difference is not understood; it may simply reflect the use of two different methods.

To confirm this result and to provide further insights into DNA binding, we also compared, by fluorescence polarization, the ability of the wt OBD and F258L mutant to bind a duplex DNA molecule containing a single GAGGC pentanucleotide (TBS; Fig 6A). As a control for specificity, a similar probe in which the T-ag binding site was inactivated by five mutations (MUT; containing 5’-AGAAT-3’ in place of 5’-GAGGC-3’) was also used. Titration of the two OBDs resulted in a dose-dependent increase in polarization (expressed as an anisotropy gain) with the TBS probe (Fig 6B), indicative of their binding to DNA. Binding of these two proteins to the control probe lacking a TBS was much weaker, confirming that both bind DNA in a sequence-specific manner. By linear regression and fitting of the data to a one-binding-site equilibrium, K_d_ values of 93 ± 21 nM and 61 ± 8 nM were obtained for the binding of the WT and F258L OBDs to the one TBS-containing probe (Fig 6C). This less than two-fold difference in K_D_ measurements confirms that the F258L substitution does not significantly alter the affinity of the JCV T-ag OBD for its pentanucleotide target sequence.

### 4. The interface formed by the F258L JCV T-ag OBD

The DNA binding studies support the conclusion that the F258L mutation does not significantly alter the structure of the DNA binding A1 and B2 loops. Therefore, we next focused on another prominent feature of the JCV T-ag OBD, the subunit-subunit interface it forms. In the previously reported wt JCV T-ag OBD crystal structures [42], we observed a relatively small interface (~550 Å^2^) between neighboring OBD molecules. The interface was formed by the A1 & B2 regions of one OBD interacting with the C-terminal pocket of a neighboring OBD; thus, the OBDs were arranged in a head-to-tail manner [42]. Furthermore, the wt JCV T-ag OBD interface was analogous to the OBD:OBD interface observed in previous SV40 crystal structures wherein the OBDs formed a crystallographic spiral having 6 molecules/turn [33, 34]. In light of the SV40 studies, we hypothesized that the interface observed in the structures of the wt JCV T-ag OBDs is similar to that formed during hexamerization of full-length T-antigen [42].

The JCV T-ag OBD containing the F258L mutation crystallized in the same space group as one of the wild type JCV OBDs, suggesting a very similar, but not identical interface as the wild type. Therefore, to refine our understanding of the defect in the F258L mutant, the differences between the interfaces formed by the wt and F258L T-ag OBDs were analyzed in detail.

#### a. Analyses of the interface formed by the JCV T-ag OBD F258L mutant

**Fig 7:**
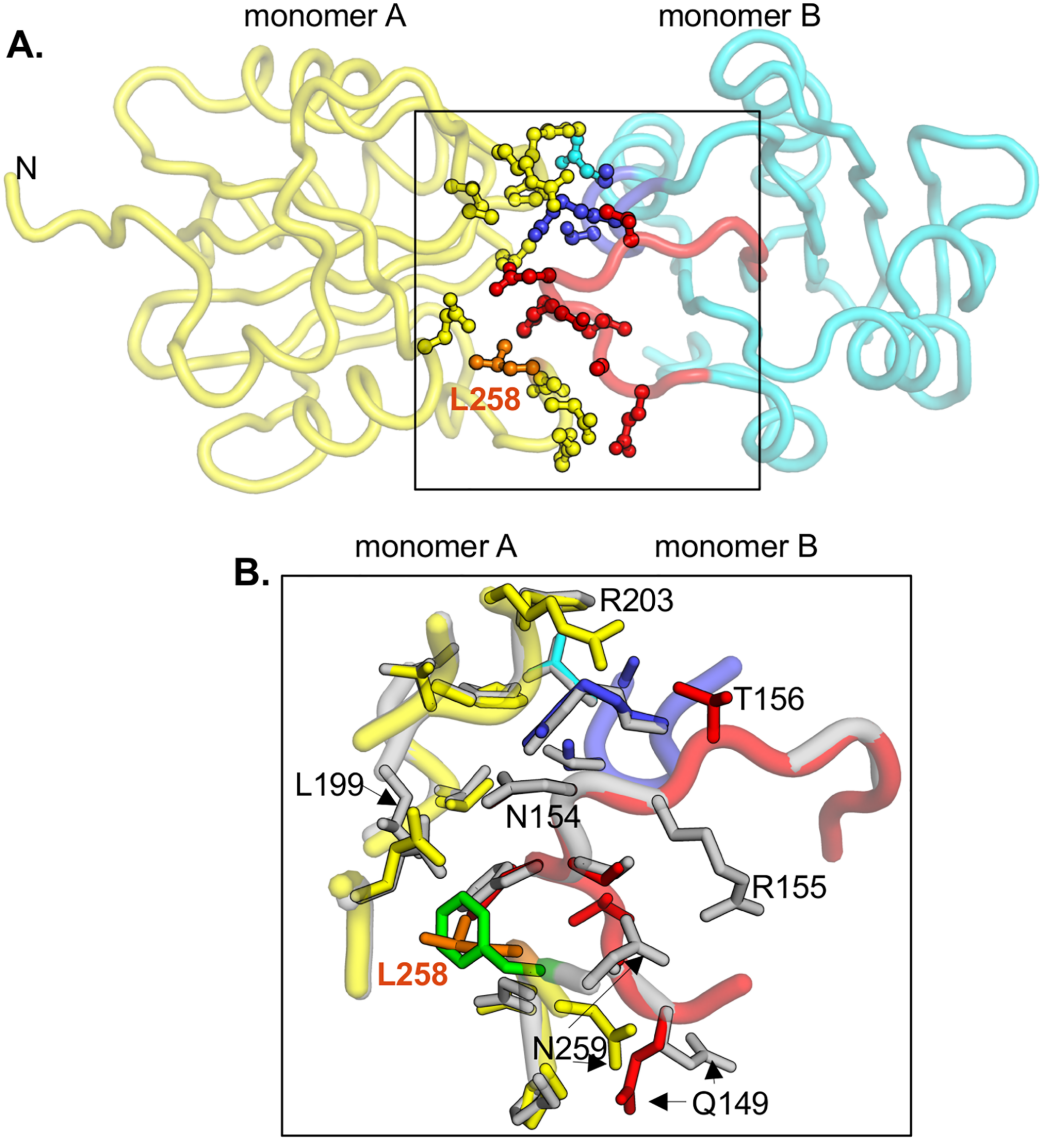
The interfaces formed by the wild type and the F258L mutant. **A.** The crystallographic interface formed by the JCV T-ag OBD F258L mutant. Monomer A is shown in yellow, while monomer B is in cyan. In monomer A, residue L258, situated in the C-terminal pocket, is presented in orange. In monomer B, residues from the A1 loop are shown in red; those from the B2 loop are in blue. Side-chains of residues involved in the interface are presented as ball and stick. **B.** Close-up of the superposition of the interfaces formed by the F258L mutant (colored as described above) and the wild type OBD interface, shown in gray. Residues that are labeled participate in interface formation and differ between the wt and F258L mutant. L258 is shown in orange, F258 in green.

The interface formed by the F258L mutant is shown in Fig 7A (monomer A in yellow, monomer B in cyan; residue L258 colored orange). As previously noted, the interface is formed when the A1/B2 loops in one monomer (red and blue residues, respectively) dock to the C-terminal pocket in a second monomer. A superposition of the interfaces present in the wt JCV OBD (both monomers in gray), and F258L mutant (colored as in Fig 7A), is shown in Fig 7B. The extent to which F258 (in green) and L258 (in orange) overlap is indicated. Close inspection reveals several subtle differences between the wt and mutant structures (e.g., differences in the positions of side chains of residues Q149 and N259).

**Fig 8:**
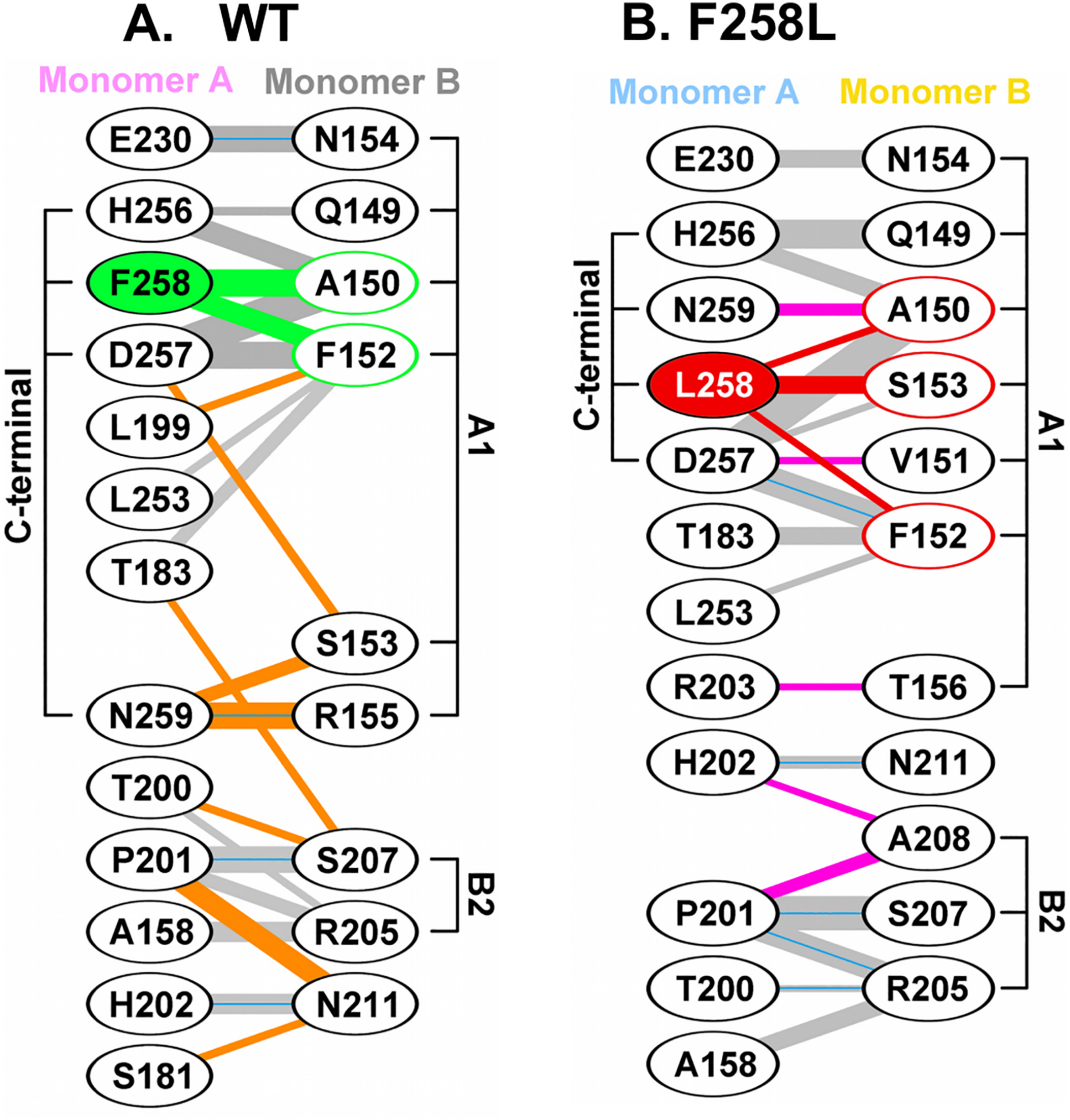
Diagrams depicting the interactions within the interfaces formed by the wild type and F258L OBDs. **A.** Interactions between monomers A and B within the wild type JCV T-ag OBD, calculated using the program PDBSUM [57]. Residues within the A1 and B2 loops, as well as C-terminal regions, are indicated. Residue F258, and the interactions it makes with the second monomer, are shown in green. Hydrogen bonds are indicated by solid blue lines while non-bonded contacts are indicated by the gray lines (the width of the line is proportional to the relative strength of the interaction). Interaction that are unique to the wild-type are in orange. **B.** A diagram depicting the interactions between monomers A and B within the F258L mutant. Residue L258, and the interactions it makes with the second monomer, are shown in red. Interactions that are unique to the F258L mutant are in pink.

An alternative comparison of the interfaces formed in the wt and F258L mutants is presented in Fig 8. Inspection of Fig 8A demonstrates that wt pocket residue F258 makes many contacts with residues in the A1 motif in the neighboring monomer. Examination of Fig 8B establishes that the A1 motif docks into the pocket present in the L258 mutant in a very similar manner. However, comparison of these two diagrams reveals that the substitution of L258 for F258 results in numerous changes. For example, the H-bond formed between N259 and R155 is lost and many non-bonded interactions made between the C-terminus and the A1 and B2 motifs are altered.

### 5. The conformation of the F258 containing C-terminal region in polyomavirus T-antigens

The studies presented in Figs 7 and 8 provide additional evidence that in the apo form of the JCV T-ag OBDs, the residue 258 containing C-terminal region plays a critical role in forming the interface with the A1/B2 loops. The A1/B2 regions are, however, initially involved in site-specific binding to DNA in the viral origin [43, 44, 65]. Moreover, DNA is known to alter the conformation of the A1 loop in SV40 T-ag (e.g., [27, 67]). These observations raised the question of whether DNA binding is also altering the conformation of the C-terminal region of polyomavirus T-ag OBDs.

**Fig 9:**
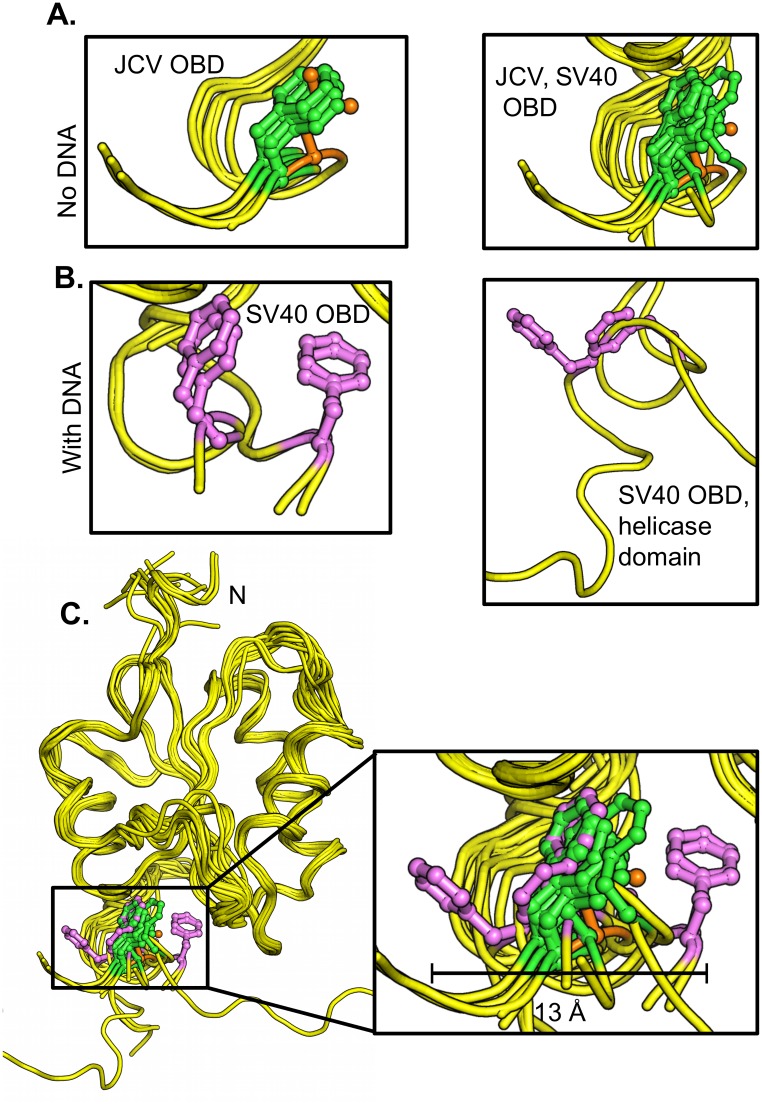
Structural based comparison of residue F258 in various polyomavirus T-ag OBDs. **A.** (Left). Superposition of the apo structures of the JCV T-ag OBDs [42], including the structure of the JCV T-ag OBD F258L mutant (F258 in green, L258 in orange) indicating the position of residue 258. (Right). Superposition of all of the apo structures of the JCV and SV40 T-ag OBDs indicating the positions of JCV T-ag residue F258 and SV40 T-ag residue F257 (colored as above). **B.** (Left). Superposition of the DNA bound forms of the SV40 OBD [27, 28, 35], showing the location of T-ag residue F257 in magenta. (Right). The location of SV40 T-ag OBD residue F257 (magenta) in the T-ag_131-627_ crystal structure [31]. **C.** Superposition of all of the above SV40 and JCV T-ag structures showing the relative locations of F257/258 (colored as above). The insert depicts a close up of the three main orientations of residue 258 (257 in SV40); the central "apo position" and the two distal orientations (the distance seperating the distal orientations is indicated (~ 13 Å). PDB IDs used in these analyses were: 1) JCV T-ag OBD apo (4NBP, 4LIF, 4LMD), 2) SV40 T-ag OBD apo (2FUF, 2IF9, 2IPR, 2ITJ, 3QK2), 3) SV40 T-ag OBD co-structures with DNA (2ITL, 2NL8, 2NTC, 4FGN, 5D9I) and 4) the SV40 T-ag_131-627_ fragment with DNA (4GDF).

A superposition of residue 258 in the apo forms of the JCV OBDs solved to date, including residue 258L (in orange), is presented in Fig 9A (left). Extending these analyses, a superposition of both JCV residue 258, and SV40 residue 257 (equivalent to JCV OBD residue F258) in all of the apo forms of the JCV and SV40 OBDs is presented in Fig 9A (right). It is apparent that in the absence of DNA, the phenylalanines, and the single leucine present in the 258L mutant, adopt a highly conserved conformation. Regarding the question of whether these structures change upon DNA binding, co-structures of the JCV T-ag OBD bound to DNA have yet to be determined. Therefore, to ascertain whether structural changes occur within the C-terminal region of the OBDs upon DNA binding, the analyses were conducted with the previously determined co-structures of the SV40 T-ag OBDs. A superposition of the co-structures of the SV40 T-ag OBDs bound to DNA ([27, 28, 35]) is presented in Fig 9B (left). Of interest, in several of the DNA bound co-structures, the position of residue F257 is altered. Moreover, the co-structure of a larger fragment of T-ag (i.e., the OBD & helicase containing T-ag_131-627_ fragment) bound to DNA has also been determined [31]. Fig 9B (right) presents just the F257 containing regions in this larger co-structure. It is apparent that in this DNA bound co-structure, residue F257 is also in a "non-apo" position. To more clearly illustrate the DNA dependent shifts in the C-terminus of the OBDs, all of the structures from JCV and SV40 T-ags were superimposed (Fig 9C; same coloring schemes as in Fig 9A and 9B). It is apparent from this figure that DNA binding causes the region around SV40 F257/ JCV F258 to become highly dynamic. Indeed, the position of F257 varies by as much as 13 Å (see insert); nearly the same distance moved by the beta-hairpin in the helicase domain as a function of ATP hydrolysis [40]. Therefore, it is concluded that DNA binding to the A1/B2 motifs alters the conformation of the C-terminal region of the SV40 T-ag OBD.

**Fig 10:**
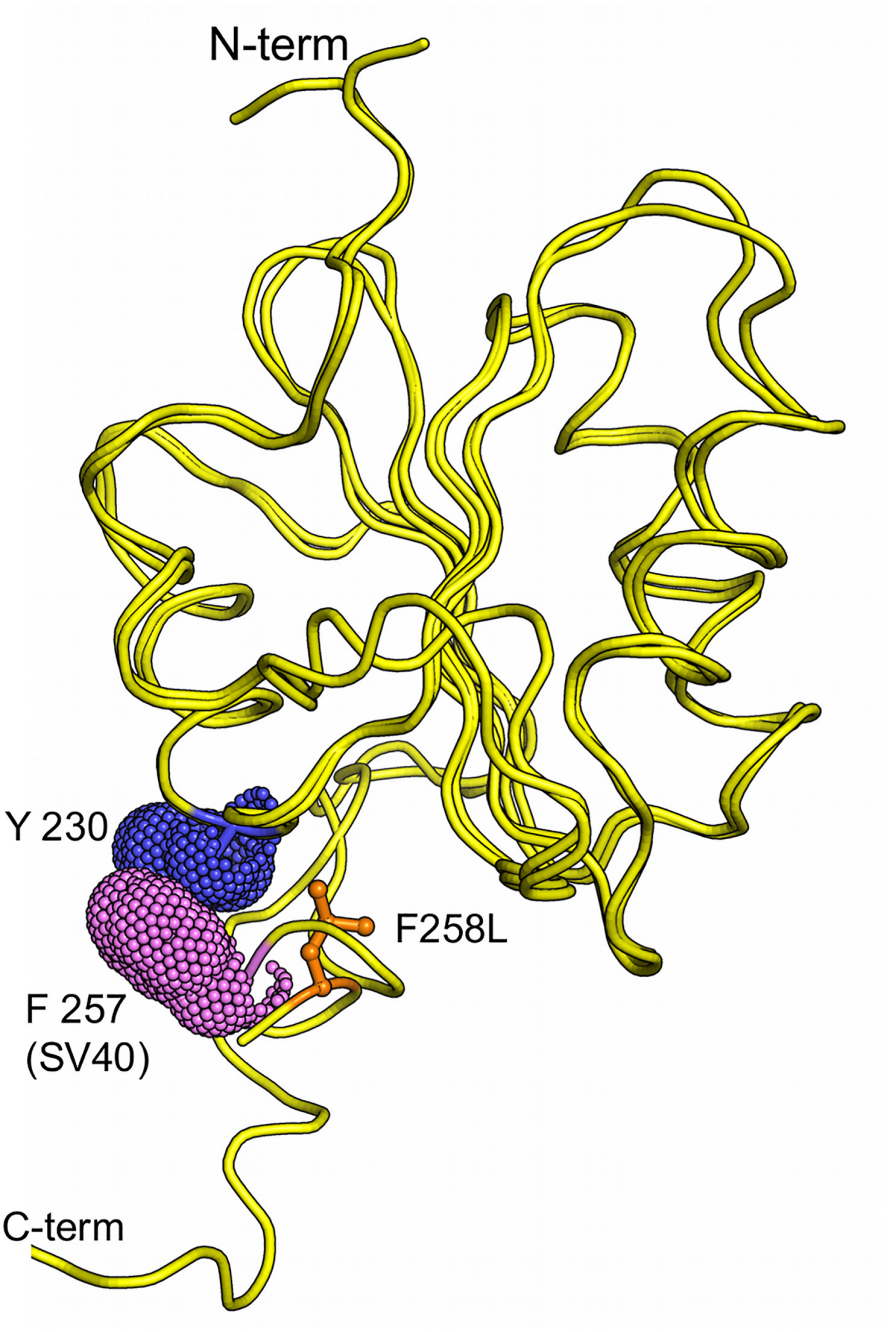
SV40 T-ag OBD residue F257 is found stacked against aromatic residues. Presented is a superposition of the SV40 T-ag co-structure (PDB ID = 4DGF) with the JCV T-ag F258L OBD mutant. SV40 T-ag residue F257 stacks against Y230, both shown as van der Waals surfaces. In contrast, JCV T-ag OBD residue L258, shown in orange, cannot partake in similar stacking interactions.

Finally, an interesting feature of the SV40 T-ag OBD co-structures solved to date is that the F257 residues in the distal orientations are often positioned opposite aromatic residues; an indication that stacking interactions may help to stabilize the individual conformations (Fig 10). Given that it lacks an aromatic ring, the F258L mutant would fail to make these stacking interactions. Therefore, in addition to perturbing the wt interface (Fig 8), the failure of the F258L mutant to form the observed stacking interactions could be an additional reason why it is defective for viral replication.

## Discussion

The F258L mutation in JCV T-ag inactivated JCV DNA replication [42]. Since a conserved feature of all human polyomavirus T-ag's is a Phe at this position, we elected to further characterize this mutation. The immunofluorescence studies established that the F258L mutation does not change the sub-cellular localization of full-length JCV T-ag. Furthermore, equal levels of wt and F258L T-ag were detected 72 hrs post-transfection [42], a finding that established that the F258L mutation does not change the stability of JCV T-ag. Therefore, to gain further insights into the function(s) of residue F258, and the C-terminal region of the T-ag OBD, we determined the crystal structure of the JCV T-ag OBD F258L mutant.

The crystallography experiments establish that the overall structures of the apo forms of the wt and F258L JCV T-ag OBDs are quite similar and that the structural consequences of the F258L mutation appear to be localized to the pocket. That the changes are localized to the pocket, and not transmitted to other regions of the JCV T-ag OBD, is supported by the observation that the F258L mutation has little to no impact on A1 and B2 loop dependent site-specific binding to duplex DNA. A related issue is whether the structural alterations resulting from the F258L mutation can be transmitted to neighboring domains of T-ag. It is noted that flexible linkers separate the polyomavirus OBDs from the neighboring J and helicase domains (e.g., [31, 32]). Therefore, it is unlikely that the structural ramifications of the F258L mutation are transmitted to other T-ag domains.

Regarding the precise changes that occur in the OBD:OBD interface as a result of the F258L mutation; pocket residue F258 normally makes contacts with A1 residues F152 and A150 (Fig 8). While L258 makes contacts to both of these residues, the interactions are different. Moreover, L258 causes disruptions to many of the non-bonded interactions and the L258 mutation also causes the loss (e.g., N259-R155) and acquisition (e.g., D257-F152) of several hydrogen bonds. Collectively, these findings establish that in the apo structure, the F258L mutation results in many subtle, but important changes to the OBD:OBD interface. It is, however, not clear if inactivation of JCV DNA replication by the F258L mutation is the direct result of changes to the interface or if other disruptions play equally important roles (e.g., the failure to stack with neighboring aromatic residues (Fig 10)).

In the apo structures of the JCV [42] and SV40 T-ag OBDs (reviewed in [26]), the positions and orientation of the F258 (F257 in SV40) residues are nearly identical (Fig 9A; (right)). In contrast, in the presence of DNA, the F257 containing C-terminal region in the SV40 T-ag OBD adopts additional orientations (Fig 9B). Thus, it is apparent that DNA binding to the A1/B2 regions alters the conformation of the C-terminal region of the SV40 T-ag OBDs. Support for this hypothesis comes from our previous NMR studies of the SV40 T-ag OBD [67]. When single-stranded poly(dT)_25_ was added to a sample of the SV40 T-ag OBD, the three regions that had large chemical shift differences were the DNA binding A1 and B2 regions, and the C-terminus. Given the extensive homology between SV40 and JCV T-ag OBDs, it is likely that DNA binding will also modulate the conformation of the C-terminus of the JCV T-ag OBD.

### 1. What are the functional consequences of the DNA dependent changes in the conformation of the C-termini of the T-ag OBD?

There are several possible functional consequences of the DNA dependent conformational changes in the OBD C-termini; one being that they play a role during T-ag assembly on the origin. Support for this hypothesis includes the finding that SV40 T-ag residue F183 is needed for oligomerization ([62, 65]; in JCV T-ag, the analogous residue (i.e., F184) sits at the base of the C-terminal OBD pocket). DNA dependent conformational changes in the OBD might also play a role in the poorly understood melting of the central or “site II” region of the core origin [68].

**Fig 11:**
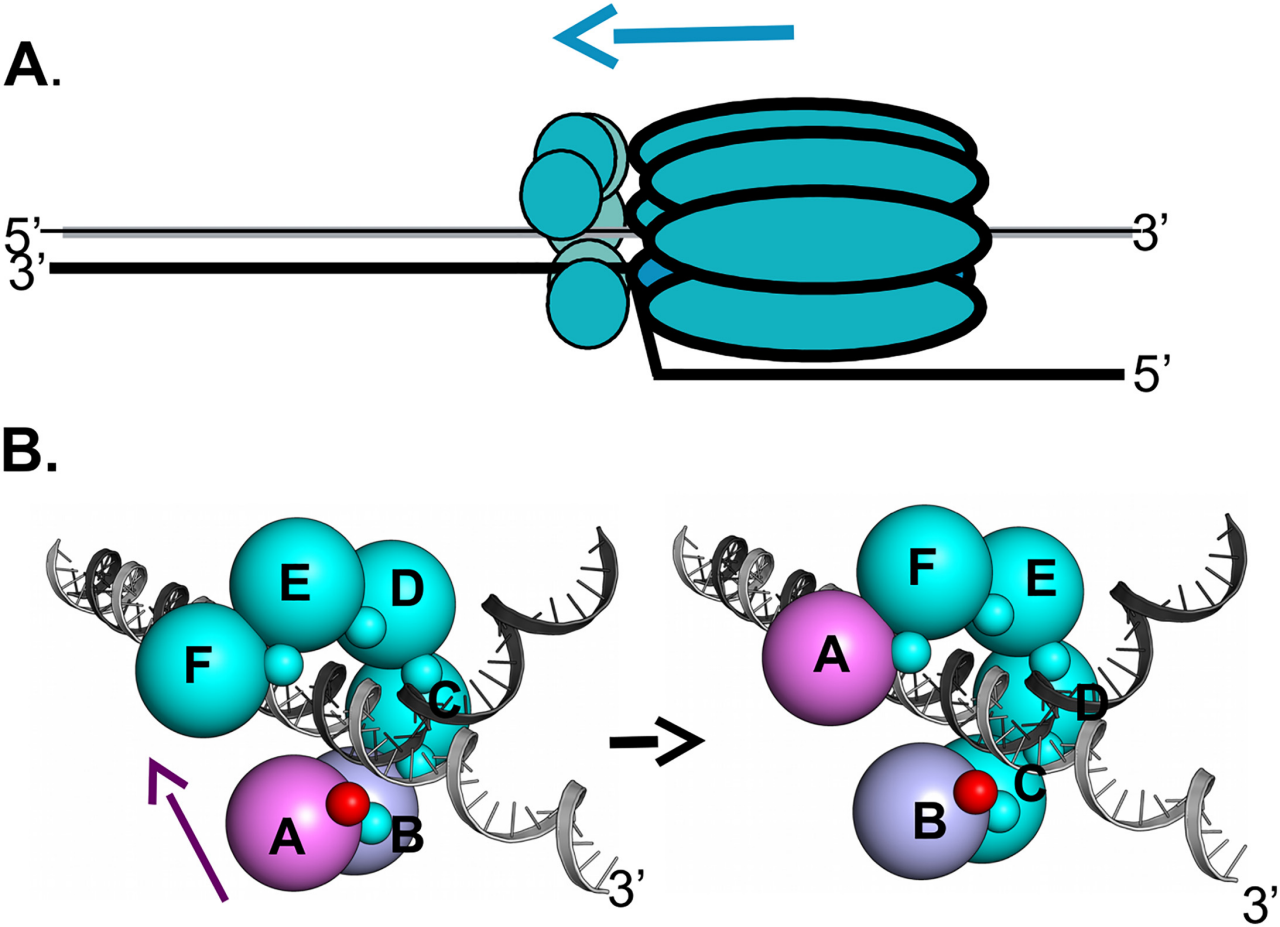
A model depicting how DNA dependent conformational changes in the C-terminus of the T-ag OBDs could regulate interface formation within T-ag hexamers. **A**. A rendering of a single T-ag hexamer assembled at a replication fork. The OBDs (small spheres) are depicted as being proximal to the forks and assembled into a hexameric spiral (reviewed in [26]). The overall 3' to 5' movement of the T-ag helicase is suggested by the blue arrow. Not shown are the flexable linkers connecting the OBDs to the helicase domains. **B**. Depiction of the proposed DNA dependent dynamics within the OBD spiral at a replication fork. The structure based model of a hexameric OBD spiral at a replication fork is adapted from previous models ([37]; reviewed in [26]). The OBDs are depicted as spheres of ~ 32 Å diameter, labeled A-F, that are situated at the center of mass of each OBD. The multifunctional A1/B2 regions are depicted as very small spheres. (Left side): In the terminal A subunit of the spiral (pink), the A1/B2 region (small red sphere), is free and thus available for interactions with DNA. The other A1/B2 regions are involved in OBD:OBD interface formation (small teal colored spheres). When the A1/B2 region in subunit A interacts with the ds/ss fork, the DNA dependent conformational changes in the F257/258 containing C-termini are induced. As a result, the interface between OBD subunits A and B (purple and light blue; respectively) is disrupted. The freed OBD subunit is then free to participate in a "hand-over-hand" movement (purple arrow) and bind its A1/B2 motif to the pocket (symbolized as a "p") in subunit F. (Right side): The A1/B2 region (small red sphere) on subunit B is now accessible. Therefore, the cycle continues when the free A1/B2 regions on subunit B engage the ds/ss DNA at the fork.

In addition, DNA dependent conformational changes in the OBD C-termini may promote DNA replication at later stages, such as when T-ag serves as the helicase at replication forks. We previously proposed that when T-ag is functioning at replication forks, that the SV40 T-ag OBDs are proximal to the ds/ss fork (Fig 11A; reviewed in [26]). Studies of the closely related papillomavirus E1 hexameric helicase also place the N-terminal DNA binding domain (DBD) at the replication fork [69–71]. Among the advantages of having the T-ag OBDs arranged at the replication fork is that as with the nonplanar DnaB spiral assembly [72], the OBD subunits could engage the ds/ss fork via a "hand-over-hand" mechanism; a process that could promote DNA unwinding. Consistent with this proposal, we previously reported that the SV40 T-ag OBDs may assemble into nonplanar hexameric spirals ([33–35]; reviewed in [26]). An additional advantage of hexameric spirals is that in one terminal monomer of the spiral the A1/B2 region is free and thus available for interactions with DNA. In contrast, were the OBDs to adopt a planar flat ring assembly, all of the A1/B2 regions would be involved in interface formation and thus unavailable to bind to DNA.

When the studies summarized above are considered in terms of our current findings they suggest a mechanism for promoting cycling of the JCV T-ag OBD monomers at the replication fork. According to this model, when the A1/ B2 regions in OBD subunit A, the terminal monomer within the spiral that is colored purple, interact with DNA at the fork (Fig 11B; left side), the DNA dependent structural changes in the C-terminus of the OBD will be induced (Fig 9C). This in turn promotes the disruption of the interface formed by the proximal pair of monomers within the spiral (subunits A & B). Consistent with this proposal, when bound to DNA the SV40 T-ag OBDs do not form "apo" like interfaces (reviewed in [26]). Once the interface is disrupted and subunit A is released from the spiral, the A1/B2 region is exposed on what had been the penultimate monomer in the spiral (subunit B (colored in blue)). Following engagement of the ds/ss forked DNA by the newly exposed A1/B2 regions on subunit B (Fig 11B; right side), the DNA dependent cycle of interface disruption will be repeated. Thus, according to the DNA dependent "interface disruption" model, the splitting apart of the OBD/OBD interface is an active process that does not depend upon the relatively inefficient thermal breakage of the interface.

### 2. Testing the "interface disruption" model and its predictions

Validation of the DNA dependent "interface disruption" model will require additional structural studies. For example, co-structures of full-length JCV T-ag or T-ag domains bound to DNA are needed to confirm the predicted DNA dependent movements within the C-termini of the JCV T-ag OBDs. Related studies are required to determine if DNA dependent movements occur in other polyomavirus OBDs. Also warranted are structural studies of the OBD:OBD interface formed in the context of T-ag hexamers. It is noted, however, that analogous DNA dependent changes in the DBD of papillomavirus E1 have not been reported [73, 74]. Why these DNA dependent changes are detected in the SV40 T-ag OBD, but not in the papillomavirus E1 DBD, remains to be determined. Nevertheless, recent experiments with E1 have established that the fork proximal origin-recognition domains play critical roles in regulating helicase activity [69] and that strand separation takes place inside E1 in a chamber N-terminal to the helicase domain [71].

In addition, the "interface disruption" model makes certain predictions that need to be tested. For example, the model suggests that upon encountering a gap in duplex DNA, the DNA dependent conformational changes in the OBD will not be induced. This would promote maintenance of the interface and possibly pausing of the T-ag helicase at the gap. Termination at gaps and nicks has been previously reported for prokaryotic (e.g.,[75, 76]) and eukaryotic (e.g., [77, 78]) hexameric helicases. However, it is not yet known if gaps and other forms of DNA damage cause the polyomavirus or papillomavirus hexameric helicases to pause. When completed, these experiments will address the generality of the DNA dependent structural changes within the C-termini of the polyomavirus OBDs and establish the functional consequences of these movements. Finally, MCM complexes are also known to form left-handed lock washer structures [79]. Thus, it will be of interest to determine if fork DNA promotes the cycling of MCM subunits.
