# Correction: Structural Based Analyses of the JC Virus T-Antigen F258L Mutant Provides Evidence for DNA Dependent Conformational Changes in the C-Termini of Polyomavirus Origin Binding Domains

**DOI:** 10.1371/journal.ppat.1005482

**Published:** 2016-02-29

**Authors:** Gretchen Meinke, Paul J. Phelan, Jong Shin, David Gagnon, Jacques Archambault, Andrew Bohm, Peter A. Bullock

Fig 11 is incorrect. The authors have provided the correct version of Fig 11 here.

**Fig 11:**
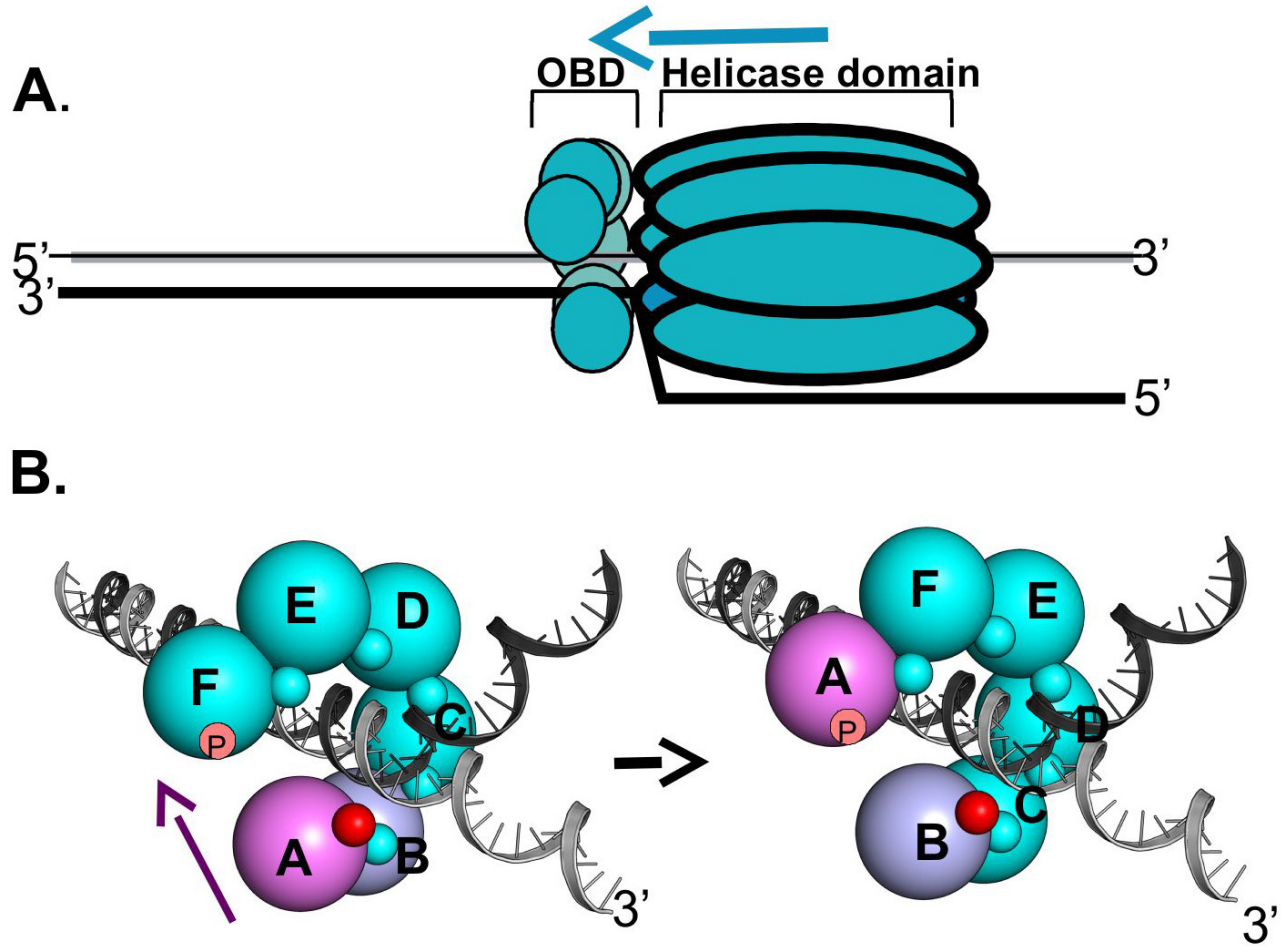
A model depicting how DNA dependent conformational changes in the C-terminus of the T-ag OBDs could regulate interface formation within T-ag hexamers. **A**. A rendering of a single T-ag hexamer assembled at a replication fork. The OBDs (small spheres) are depicted as being proximal to the forks and assembled into a hexameric spiral (reviewed in [26]). The overall 3' to 5' movement of the T-ag helicase is suggested by the blue arrow. Not shown are the flexable linkers connecting the OBDs to the helicase domains. **B**. Depiction of the proposed DNA dependent dynamics within the OBD spiral at a replication fork. The structure based model of a hexameric OBD spiral at a replication fork is adapted from previous models ([37]; reviewed in [26]). The OBDs are depicted as spheres of ~ 32 Å diameter, labeled A-F, that are situated at the center of mass of each OBD. The multifunctional A1/B2 regions are depicted as very small spheres. (Left side): In the terminal A subunit of the spiral (pink), the A1/B2 region (small red sphere), is free and thus available for interactions with DNA. The other A1/B2 regions are involved in OBD:OBD interface formation (small teal colored spheres). When the A1/B2 region in subunit A interacts with the ds/ss fork, the DNA dependent conformational changes in the F257/258 containing C-termini are induced. As a result, the interface between OBD subunits A and B (purple and light blue; respectively) is disrupted. The freed OBD subunit is then free to participate in a "hand-over-hand" movement (purple arrow) and bind its A1/B2 motif to the pocket (symbolized as a "p") in subunit F. (Right side): The A1/B2 region (small red sphere) on subunit B is now accessible. Therefore, the cycle continues when the free A1/B2 regions on subunit B engage the ds/ss DNA at the fork.
